# Author Correction: Charge-state lifetimes of single molecules on few monolayers of NaCl

**DOI:** 10.1038/s41467-023-41399-z

**Published:** 2023-09-08

**Authors:** Katharina Kaiser, Leonard-Alexander Lieske, Jascha Repp, Leo Gross

**Affiliations:** 1 IBM Research Europe—Zurich, Säumerstrasse 4, 8803 Rüschlikon, Switzerland; 2 Department of Physics, University of Regensburg, Universitätsstraße 31, 93053 Regensburg, Germany; 3 Present Address: Université de Strasbourg, CNRS, IPCMS, UMR 7504, F-67000 Strasbourg, France

**Keywords:** Surfaces, interfaces and thin films, Nanoparticles

Correction to: *Nature Communications* 10.1038/s41467-023-40692-1, published online 17 August 2023

The original version of this Article contained an error in Fig. 1b, in which the labels of the chemical structures ‘ZnPc’ and ‘H_2_Pc’ had been interchanged. This has been corrected in both the PDF and HTML versions of the Article.

