# Supplementary material for: Charge-state lifetimes of single molecules on few monolayers of NaCl
Source: Nat Commun. 2023 Aug 17;14:4988. doi: 10.1038/s41467-023-40692-1 (PMC10435478; doi:10.1038/s41467-023-40692-1)
Supplement: Supplementary file 1 — Supplementary Information for Charge-state lifetimes of single molecules on few monolayers of NaCl [file 41467_2023_40692_MOESM1_ESM.pdf]

**Supplementary Information for**

# Charge-state lifetimes of single molecules on few monolayers of NaCl

Katharina Kaiser*[1] #, Leonard-Alexander Lieske[1], Jascha Repp[2], Leo Gross*[1]

[1] IBM Research Europe – Zurich, Säumerstrasse 4, 8803 Rüschlikon, Switzerland
[2] Department of Physics, University of Regensburg, Universitätsstraße 31, 93053 Regensburg, Germany
# Present address: Université de Strasbourg, CNRS, IPCMS, UMR 7504, F-67000 Strasbourg, France

*Corresponding authors. E-mail: katharina.kaiser@ipcms.unistra.fr, LGR@zurich.ibm.com

Contents:

Supplementary Note 1. Additional constant-height current maps

Supplementary Note 2. Distance and voltage dependence of the charge-state lifetime

Supplementary Note 3. Decharging from the substrate

Supplementary Note 4. Extraction of effective NaCl barrier heights

Supplementary Note 5. Estimation of valence band maximum and conduction band minimum

Supplementary references

**Supplementary Note 1. Additional constant-height current maps**

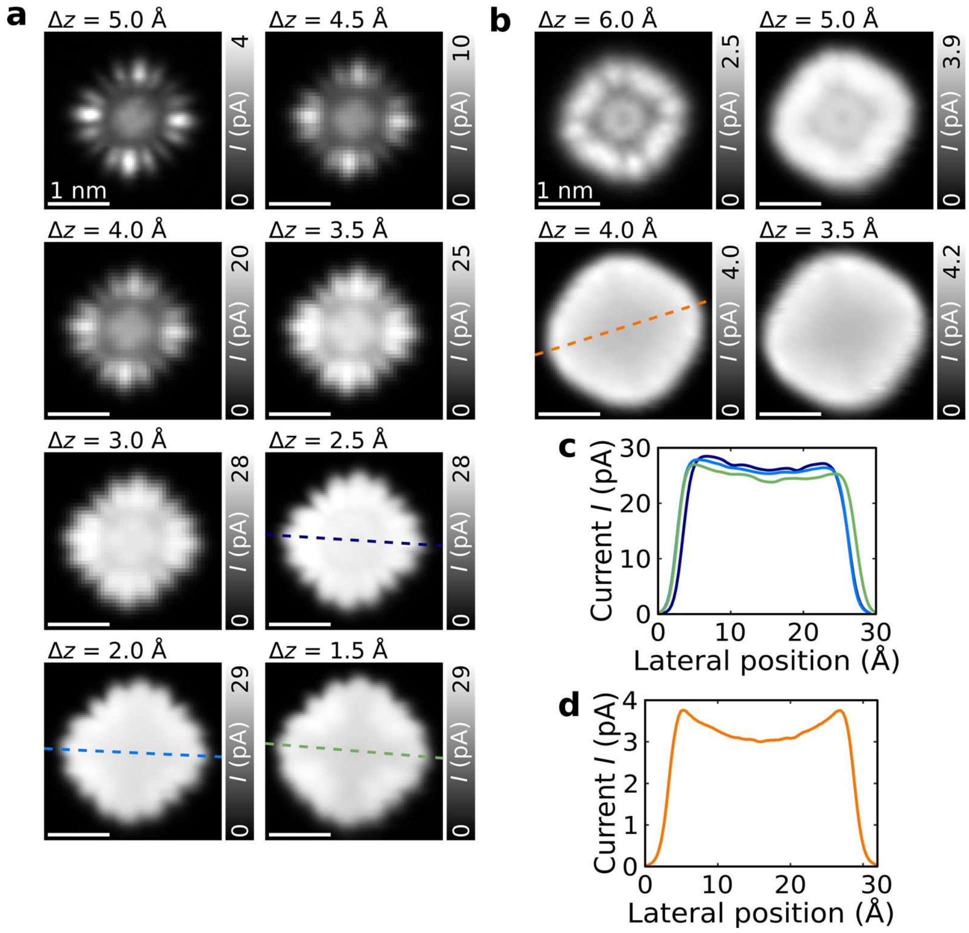


**Supplementary Figure 1**: **Current-maps as a function of tip height on ZnPc.** Constant height current maps at different tip-sample distances on ZnPc adsorbed on **a** 4 ML NaCl/Au(111), recorded at the PIR at $V$ = -1.2 V and **b** 5 ML NaCl/Au(111), recorded at the NIR at $V$ = 2.2 V. The line profiles in **c** and **d** correspond to current traces along the respectively colored dashed lines at $\Delta z$ = 2.5 Å, 2 Å and 1.5 Å in **a** as well as $\Delta z$ = 4 Å in **b**. $\Delta z$ is given with respect to the STM setpoint of $V$ = -1.2 V, $I$ = 0.5 pA above the bare NaCl surface in **a** and $V$ = 2.2 V, $I$ = 0.5 pA in **b**. The scale bars correspond to 1 nm.

Supplementary Figure 1 shows additional constant-height current maps recorded on ZnPc on 4 and 5 ML NaCl/Au(111). To ensure that the probed ionic resonance (PIR or NIR) is accessible at all probed tip heights and NaCl thicknesses, we used bias voltages $V$ slightly above (below) the peak position of NIR (PIR) in d$I$/d$V$. This is further detailed in Supplementary Note 2. In the constant-height STM images in the regime of current saturation (see linescans in Suppl. Fig. 1c, d), we observe a slightly larger saturation current at the rim of the molecule compared to its center. This effect probably results from a different screening of the tip's electric field by the molecule at different tip positions, slightly affecting the tunnel rates through the NaCl barrier.

**Supplementary Note 2. Distance and voltage dependence of the charge-state lifetime**

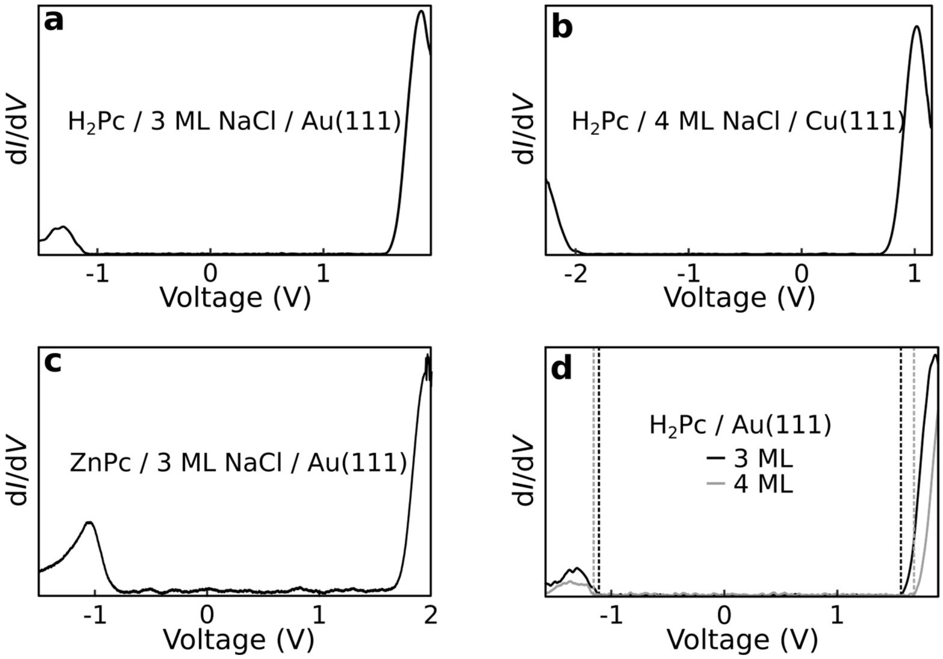


**Supplementary Figure 2**: **d*I*/d*V*(*V*) recorded on different systems. a** $H_2Pc$ on 3 ML NaCl/Au(111) (setpoint $I$ = 0.5 pA, $V$ = 2 V, $\Delta z$ = -1 Å). **b** $H_2Pc$ on 4 ML NaCl/Cu(111) (setpoint $I$ = 0.5 pA, $V$ = 1.6 V, $\Delta z$ = -1 Å). **c** ZnPc on 3 ML NaCl/Au(111). **d** Comparison of $H_2Pc$ on 3 ML and on 4 ML NaCl/Au(111) (setpoint $I$ = 0.5 pA, $V$ = 2 V, $\Delta z$ = -1 Å). The onsets of PIR and NIR are indicated by the dashed lines.

The voltage drop across the tip-sample junction is composed of the voltage drop over the vacuum gap (between tip and molecule) and the one over the NaCl film (between molecule and metal substrate). The ratio of these two voltage drops depends on the exact geometry, *i.e.*, the tip-molecule distance $z$, the tip position, the thickness of the NaCl film $d_{NaCl}$, and the tip-radius. Following the estimation given in ref. [1], we estimate the relative voltage drop over NaCl to be in the range of 10-20% of the total applied bias voltage for the geometries present in this study.

Upon applying a bias, the voltages of the molecular ion resonances are shifted with respect to the zero bias condition by the voltage drop across the NaCl film. As a consequence, changes in $z$ or $d_{NaCl}$ directly affect the voltages of the ion resonances. To avoid shifting of the molecular ion resonances outside the voltage window when changing the lever arm ($z$ or $d_{NaCl}$), we typically used absolute bias voltages slightly larger (up to a few 100 mV larger) then the corresponding peak positions in d$I$/d$V$, $V_{PIR}$ and $V_{NIR}$. One example for the shift due to the changed lever arm is shown in Suppl. Fig. 2d, where we show d$I$/d$V$($V$)-curves recorded atop $H_2Pc$ adsorbed on 3 and 4 ML of NaCl on Au(111). The peaks and onsets of PIR and NIR shift to higher absolute values on 4 ML NaCl, as a result of the larger relative voltage drop across NaCl on 4 ML compared to 3 ML.

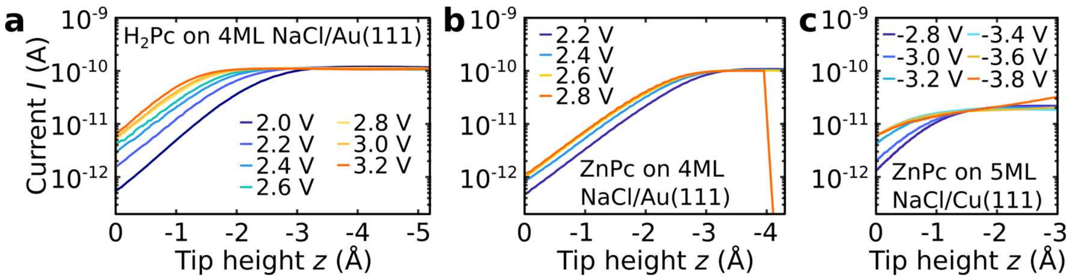


**Supplementary Figure 3: *I*(*z*) spectroscopy at different *V*.** **a** *I*(z) at different positive bias voltages atop a lobe of an $H_2Pc$ and **b** ZnPc adsorbed on 4 ML NaCl/Au(111). In **b**, the ZnPc molecule dislocated at $V$ = 2.8 V at about $z$ = -4 Å. **c** *I*(*z*) at different negative bias voltages atop one lobe of a ZnPc adsorbed on 5ML NaCl on Cu(111). Here, $z$ corresponds to the tip-height offset $\Delta z$ when the STM feedback was opened, *i.e.*, at the position the spectrum was recorded at $I$ = 0.5 pA, and $V$ = 2.0 V (**a**), $V$ = 2.2 V (**b**), and $V$ = -2.8 V (**c**).

A similar trend is expected for decreasing $z$ (*i.e.*, a decrease in $z$ will lead to an increase in voltage drop across NaCl and thus an up-shift of the absolute values of $V_{PIR}$ and $V_{NIR}$) [2]. Unaffected by the changed lever arm, the saturation current, *i.e.*, the charge-state lifetime, does not vary with decreasing $z$ (see, *e.g.*, Fig. 2b in the main text). In addition, we recorded bias dependent *I(z)* curves (Suppl. Fig. 3), showing that, also independent of the applied bias voltage, the charge-state lifetime remains the same. This indicates that the charge-state lifetime, and with it the barrier height for the corresponding tunneling process through NaCl, does not vary significantly as a function of the voltage drop across NaCl.

However, for significantly increased voltages, see Suppl. Fig. 3c at $V$ = 3.8 V, the saturation current becomes affected by the increased voltage. This indicates that for ZnPc on Cu(111) for $V \leq -3.8$ V additional tunnel channels become accessible. Supplementary Figure 4 shows higher-lying states that could potentially contribute to the overall tunnel current at elevated bias voltages (*i.e.*, -3.8 V on Cu(111), Suppl. Fig. 3c, and -2.5 V on Au(111), Fig. 5) in an energy diagram including many-body transitions. Note that here we do not discuss transitions to states that become directly accessible from $S_0$ in a single-electron tunneling event (faded arrows in Suppl. Fig. 4) for the following reason: The increase in current above $I_{sat}$ and change in contrast in c.h. STM only appears at small $z$, *i.e.*, sets in only at a large tunnel rate between tip and molecule. This indicates that the underlying process is of higher order (*i.e.*, a two- or more-electron process) and involves tunneling into a transient state of the molecule (*e.g.*, $T_1$ or $D_0^+$) and hence only becomes accessible if charge transfer from the tip happens at comparable or faster time scales than the depopulation of the involved transient state.

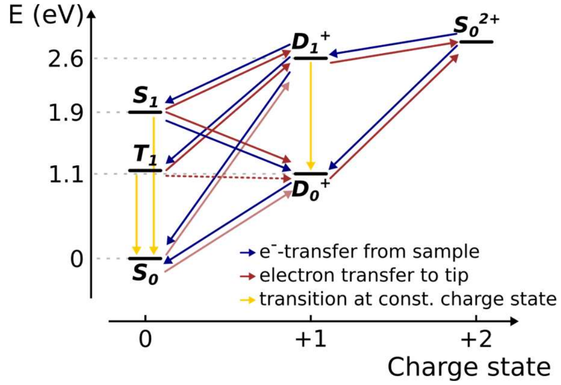


**Supplementary Figure 4**: **Energy diagram of ZnPc on Au(111).** Energy diagram including higher-lying states and possible many-body transitions that become accessible at increased absolute voltage values for ZnPc adsorbed on a few ML NaCl on Au(111). Blue arrows indicate charge-state transitions by tunneling between molecule and sample (through the NaCl barrier), and red arrows by tunneling between molecule and tip (through the vacuum barrier). Single-electron transitions from the neutral ground state $S_0$ (faded red lines) are not considered for the observed increase in current at small z. Yellow arrows indicate transitions that do not involve a change in charge state, such as radiative transitions. The dashed red arrows indicate charge-state transitions in which electrons are removed from higher-lying orbitals (the LUMO of the neutral molecule). These transitions could lead to the observed contrast in constant-height STM images at $V$ = -2.5 V and small tip height. Note that this diagram is simplified and does not contain additional, even higher-lying excited states of the system.

At elevated bias voltages and for small *z,* charge transfer from tip to molecule could thus lead to transitions to $S_0^{2+}$, from which transitions to $D_1^+$ are possible, as indicated in Suppl. Fig. 4, which can rationalize the observed increase in saturation current. Furthermore, the observation of the shape and symmetry of the LUMO density could relate to charge state transitions from the tip that involve higher-lying orbitals, as indicated by the red dashed lines in Suppl. Fig. 4. The observed increased tunnel current in Fig. 5, which resembles the shape of the LUMO, could be caused, for example, by alternating charge transitions between the $D_1^+$ and $S_0^{2+}$ states.

**Supplementary Note 3. Decharging from the substrate**

Figure 4 in the main text schematically depicts, in a single-electron picture, the molecular energy levels of ZnPc/$H_2$Pc after charging from the tip. Note that the single-electron picture is used here to depict the level alignment of the channels with respect to, *e.g.*, valence band (VB), conduction band (CB) and interface state (IS). Although the energy levels derive from the neutral molecule's HOMO and LUMO, their energetic positions are different from the neutral molecule's levels because of, *e.g.*, Coulomb interaction, the lifted spin degeneracy, and reorganization [1,3]. The exact energetic positions of these levels, corresponding to transiently charged states, cannot be probed in our experiment. However, we can estimate the energetic position of the cation's (anion's) singly unoccupied (occupied) frontier orbital with respect to the tip's electrochemical potential, based on the positions of the PIR (NIR) peak in d$I$/d$V$ and estimating the reorganization energy. From these levels (SOMO of anion at NIR and SUMO of cation at PIR), we construct the levels corresponding to the transitions to $S_1$ and $T_1$ states by using reported energies of luminescence and phosphorescence, respectively. The positions of the lowest unoccupied level at NIR and the highest occupied level at PIR are estimated from experiments in which molecules were doubly negatively charged on thick NaCl films [4], with consideration of the lever arm [3]. The charging energy $E_{\text{charge2}}$ for doubly charged states, corresponding to the additional energy needed from the single charging event to double charging, is estimated here as $E_{\text{charge2}}$ = 1.2 eV for both dianions and dications.

In addition, to corroborate the accessibility of possible channels to excited states in the neutralization step, we compared our conclusions to previous STML experiments on these systems. If a molecule shows luminescence in STML at PIR or NIR (on a given surface) the transition from the corresponding charged $D_0$ state back to the neutral charge state can entail the transition to $S_1$ [5–8]. Hence, if at a given bias voltage luminescence can be observed for the system, the channel to the $S_1$ state is accessible for the neutralization of the molecule.

Based on these arguments, we sketch in Fig. 4 the energetic positions of the molecular single-electron energy levels of the charged molecule with respect to tip and sample states, taking into account several literature values and observations from STML experiments, indicating possible pathways for the neutralization of the singly charged systems.

Supplementary Figure 5 depicts the quantities that were taken into account for the level alignment presented in Fig. 4 in the main text, using the examples of the anion at bias voltages that correspond to the NIR (Suppl. Fig. 5a) and the cation at voltages corresponding to the PIR (Suppl. Fig. 5b) on Au(111). The sample voltages for these conditions, *i.e.*, at NIR and at PIR, correspond to the respective peaks in d$I$/d$V$. As a result of reorganization, the anion's (cation's) energy levels are shifted down (up) by the reorganization energy $E_{\text{reorg}}$ with respect to the corresponding energy levels of the neutral molecule. We assume here a reorganization energy of $E_{\text{reorg}}$ = 0.4 eV. The energy levels are broadened due to electron-phonon coupling [2,9]; the total peak width, being the energy range within which tunneling into the molecular orbitals is appreciable in the experiment, is here assumed to be $w$ = 0.6 eV. The energetic difference between the molecular levels are associated with the transition energies of the $S_0$-$S_1$ and $S_0$-$T_1$ transitions, respectively.

The energies for the $S_1$-$S_0$ transition in $H_2$Pc and ZnPc are 1.81 eV and 1.89 eV, respectively [5,10]. $T_1$ energies of 1.24 eV for $H_2$Pc and 1.14 eV for ZnPc have been reported for molecules in chloronaphthalene solution [11,12]. Note, however, that the presence of the substrate and the tip is known to shift the energies of optical transitions with respect to the values in solution [13,14], and phosphorescence from $T_1$ of $H_2$Pc or ZnPc in STML has not been reported as of now.

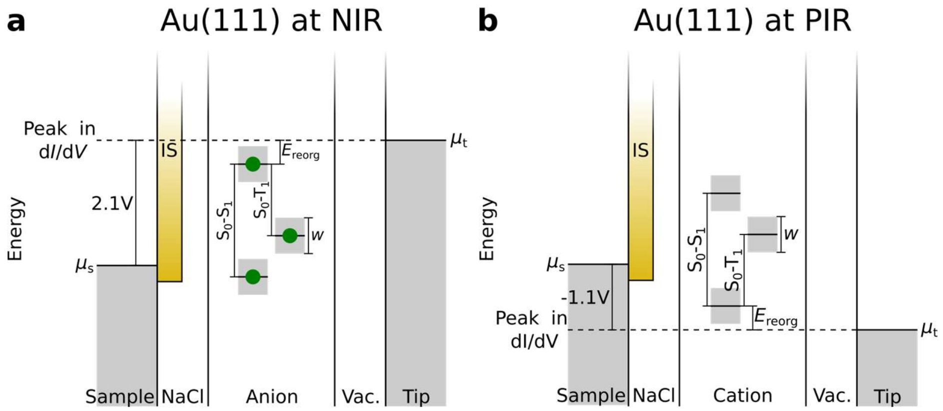


**Supplementary Figure 5**: **Energetic alignment of ionic energy levels in the double-barrier tunnel junction.** Sketch depicting the estimated quantitative alignment of the anionic **a** and cationic **b** energy levels with respect to sample and tip states. The assumed values for reorganization energy are $E_{reorg}$ = 0.4 eV, charging energy for doubly charged states $E_{charge2}$ = 1.2 eV, and linewidth of the energy levels $w$ = 0.6 eV, indicated by the grey shaded areas around the molecular levels. The energetic difference between the molecular levels are associated with the transition energies of the $S_0$-$S_1$ and $S_0$-$T_1$ transitions, respectively. The onset of the IS for NaCl/Au(111) is $V$ = -0.27 V. All shown energy differences are to scale.

Note that for the PIR on Au(111), the applied bias voltages are close to being sufficient to facilitate the formation of the $T_1$ upon neutralization from the substrate. However, the exact energies of the $T_1$-$S_0$ transition for $H_2$Pc and ZnPc adsorbed on an ultrathin insulating film atop a metal substrate are not known. We tentatively excluded the tunnel channel for $T_1$ formation in Fig. 4d based on the energetic arguments given above. However, it cannot be fully ruled out because of the uncertainty of the energy of the $T_1$-$S_0$ transition and the significant level broadening on NaCl.

The conduction band minimum (CBM) and valence band maximum (VBM) are indicated in Fig. 4 to highlight the need to consider the NaCl's band structure for tunneling. References [15–17] suggest that the CBM is roughly aligned with the vacuum level while the band gap is at least 8 eV. The NaCl film lowers the metal work function, *i.e.*, 4.9 eV for Cu(111) and 5.3 eV for Au(111), by about 1 eV [18,19]. Thus, the band gap ranges from around 4 – 5 eV below the electrochemical potential of the sample to around 3.5 – 4.5 eV above.

**Supplementary Note 4. Extraction of effective NaCl barrier heights**

For a single-barrier tunnel junction, the tunnel current as a function of distance $z$ is given by

$$I(z) = I_0 \cdot \mathrm{e}^{-2\kappa z} \qquad (1)$$

The exponent is determined by $\kappa = \frac{\sqrt{2 \mathrm{m_e} \phi_{\mathrm{eff}}}}{\hbar}$, with the electron mass $m_e$ and the effective barrier height $\Phi_{\mathrm{eff}}$. For tunneling between the molecule and metallic substrate, the effective barrier height for tunneling through the NaCl film $\Phi_{\mathrm{NaCl}}$ can be determined from the exponent of the $I_{\mathrm{sat}}(d_{\mathrm{NaCl}})$ dependence, *i.e.*, the slope $m$ of $\ln(I_{\mathrm{sat}}(d_{\mathrm{NaCl}}))$.

We determined slopes $m$ and effective NaCl barrier heights $\Phi_{\mathrm{NaCl}}$ at both ion resonances for ZnPc and $H_2$Pc adsorbed on NaCl on Au(111) and Cu(111). The results are summarized in the following Suppl. Table 1. Note that, for $H_2$Pc on Au(111) and ZnPc on Cu(111), the saturation current could only be recorded on two different NaCl film thicknesses and hence, the values determined for these systems have a larger uncertainty.

| | $m$ at PIR ($\mathrm{ML}^{-1}$) | $m$ at NIR ($\mathrm{ML}^{-1}$) | Increase of $\tau_d$ per ML NaCl at PIR | Increase of $\tau_d$ per ML NaCl at NIR | $\Phi_{\mathrm{NaCl}}$ at PIR (eV) | $\Phi_{\mathrm{NaCl}}$ at NIR (eV) |
|---|---|---|---|---|---|---|
| ZnPc/Au(111) | -3.4 ± 0.2 | -3.3 ± 0.2 | x 30 ± 6 | x 28 ± 4 | 1.3 ± 0.2 | 1.3 ± 0.1 |
| $H_2$Pc/Au(111) | -3.5 ± 0.4 | -3.2 ± 0.3 | x 32 ± 11 | x 25 ± 6 | 1.4 ± 0.3 | 1.2 ± 0.2 |
| ZnPc/Cu(111) | -2.8 ± 0.2 | -2.9 ± 0.2 | x 17 ± 4 | x 19 ± 4 | 0.9 ± 0.2 | 1.0 ± 0.2 |
| $H_2$Pc/Cu(111) | -2.9 ± 0.1 | -2.9 ± 0.4 | x 17 ± 2 | x 17 ± 6 | 0.9 ± 0.1 | 0.9 ± 0.2 |

**Supplementary Table 1**: Summary of slopes $m$, increase in charge-state lifetime per additional ML NaCl, and corresponding effective NaCl barrier height for ZnPc and $H_2$Pc adsorbed on Cu(111) and Au(111) and recorded at PIR and NIR.

The saturation current was determined with the fitting procedure discussed in the manuscript. The corresponding uncertainty is estimated based on the standard deviation of the data points.

The uncertainty margin of the slopes was determined from the linear fit in the case of ZnPc/Au(111) and $H_2$Pc/Cu(111). For $H_2$Pc/Au(111) and ZnPc/Cu(111), where the saturation current was only measured for two different NaCl film-thicknesses, this uncertainty was determined from the standard deviation of the measured saturation current. The number $N$ of independent measurements per data point are listed in the following Suppl. Table 2.

| | Au(111), NIR | | | Au(111), PIR | | | Cu(111), NIR | | | Cu(111), PIR | | |
|---|---|---|---|---|---|---|---|---|---|---|---|---|
| | *N* on 3 ML | *N* on 4 ML | *N* on 5 ML | *N* on 3 ML | *N* on 4 ML | *N* on 5 ML | *N* on 3 ML | *N* on 4 ML | *N* on 5 ML | *N* on 3 ML | *N* on 4 ML | *N* on 5 ML |
| ZnPc | 1 | 7 | 5 | 5 | 28 | 22 | 0 | 11 | 1 | 0 | 18 | 4 |
| $H_2$Pc | 7 | 21 | 0 | 12 | 12 | 0 | 2 | 4 | 1 | 1 | 2 | 2 |

**Supplementary Table 2**: Number $N$ of independent measurements per data point in the $I_{\mathrm{sat}}(d_{\mathrm{NaCl}})$ plot in Fig. 3 in the main text.

**Supplementary Note 5. Estimation of valence band maximum and conduction band minimum**

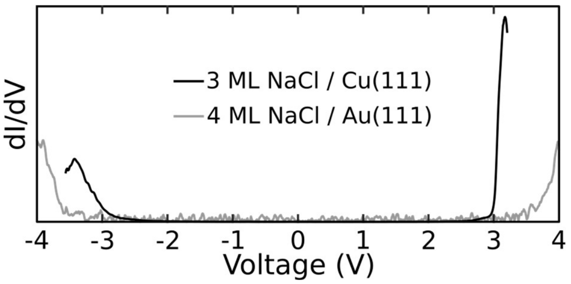


**Supplementary Figure 6**: **d*I*/d*V*(*V*) of NaCl.** d*I*/d*V*(V) on 3 ML NaCl/Cu(111) (black curve, setpoint *V* = -0.2 V, *I* = 2 pA, *Δz* = 2 Å) and 4 ML NaCl/Au(111) (grey curve, setpoint *V* = -5 V, *I* = 2 pA, *Δz* = 0 Å). The curves were obtained by numerical differentiation of *I*(*V*) curves.

Supplementary Figure 6 shows d*I*/d*V*(*V*) spectra recorded atop the bare NaCl surface for different underlying metal substrates. Note that the measured apparent onsets of tunnelling depend on the tip-NaCl distance and might relate to image potential states [20,21] and only serve as a rough lower bound for the band onsets. The combination of refs. [15–17] indicate that the CBM is located close to the vacuum level and that even for films of only few atomic layers the band gap of NaCl is (almost) fully developed, *i.e.*, about 8.5 eV. The NaCl film lowers the metal work function, *i.e.*, 4.9 eV for Cu(111) and 5.3 eV for Au(111), by about 1 eV [18,19]. We estimate that the band gaps, that is VBM and CBM, should extend from roughly 4 - 5 eV below the Fermi level to roughly 3.5 - 4.5 eV above it.

**Supplementary references**


[1] S. Fatayer, B. Schuler, W. Steurer, I. Scivetti, J. Repp, L. Gross, M. Persson, and G. Meyer, *Reorganization Energy upon Charging a Single Molecule on an Insulator Measured by Atomic Force Microscopy*, Nat. Nanotechnol. **13**, 376 (2018).

[2] J. Repp, G. Meyer, S. Paavilainen, F. E. Olsson, and M. Persson, *Scanning Tunneling Spectroscopy of Cl Vacancies in NaCl Films: Strong Electron-Phonon Coupling in Double-Barrier Tunneling Junctions*, Phys. Rev. Lett. **95**, 225503 (2005).

[3] S. Fatayer, F. Albrecht, I. Tavernelli, M. Persson, N. Moll, and L. Gross, *Probing Molecular Excited States by Atomic Force Microscopy*, Phys. Rev. Lett. **126**, 176801 (2021).

[4] S. Fatayer, F. Albrecht, Y. Zhang, D. Urbonas, D. Peña, N. Moll, and L. Gross, *Molecular Structure Elucidation with Charge-State Control*, Science **365**, 142 (2019).

[5] B. Doppagne, M. C. Chong, H. Bulou, A. Boeglin, F. Scheurer, and G. Schull, *Electrofluorochromism at the Single-Molecule Level*, Science **361**, 251 (2018).

[6] K. Miwa, H. Imada, M. Imai-Imada, K. Kimura, M. Galperin, and Y. Kim, *Many-Body State Description of Single-Molecule Electroluminescence Driven by a Scanning Tunneling Microscope*, Nano Lett. **19**, 2803 (2019).

[7] S. Jiang, T. Neuman, R. Bretel, A. Boeglin, F. Scheurer, E. Le Moal, and G. Schull, *Many-Body Description of STM-Induced Fluorescence of Charged Molecules*, Phys. Rev. Lett. **130**, 126202 (2023).

[8] T.-C. Hung, R. Robles, B. Kiraly, J. H. Strik, B. A. Rutten, A. A. Khajetoorians, N. Lorente, and D. Wegner, *Bipolar Single-Molecule Electroluminescence and Electrofluorochromism*, arXiv:2210.11118.

[9] J. Repp, G. Meyer, S. M. Stojković, A. Gourdon, and C. Joachim, *Molecules on Insulating Films: Scanning-Tunneling Microscopy Imaging of Individual Molecular Orbitals*, Phys. Rev. Lett. **94**, 026803 (2005).

[10] H. Imada, K. Miwa, M. Imai-Imada, S. Kawahara, K. Kimura, and Y. Kim, *Real-Space Investigation of Energy Transfer in Heterogeneous Molecular Dimers*, Nature **538**, 364 (2016).

[11] J. McVie, R. S. Sinclair, and T. G. Truscott, *Triplet States of Copper and Metal-Free Phthalocyanines*, J. Chem. Soc., Faraday Trans. 2 **74**, 1870 (1978).

[12] P. S. Vincett, E. M. Voigt, and K. E. Rieckhoff, *Phosphorescence and Fluorescence of Phthalocyanines*, The Journal of Chemical Physics **55**, 4131 (1971).

[13] A. Rosławska, T. Neuman, B. Doppagne, A. G. Borisov, M. Romeo, F. Scheurer, J. Aizpurua, and G. Schull, *Mapping Lamb, Stark, and Purcell Effects at a Chromophore-Picocavity Junction with Hyper-Resolved Fluorescence Microscopy*, Phys. Rev. X **12**, 011012 (2022).

[14] J. Kerfoot et al., *Substrate-Induced Shifts and Screening in the Fluorescence Spectra of Supramolecular Adsorbed Organic Monolayers*, J. Chem. Phys. **149**, 054701 (2018).

[15] Fölsch, Stefan, Elektronenspektroskopische Untersuchungen Zur H 2 O-Adsorption Auf Der NaCl(100) Oberfläche, University Hannover, 1991.

[16] F. J. Himpsel and W. Steinmann, *Angle-Resolved Photoemission from the NaCl (100) Face*, Phys. Rev. B **17**, 2537 (1978).

[17] S.-F. Tsay and D.-S. Lin, *Atomic and Electronic Structures of Thin NaCl Films Grown on a Ge(001) Surface*, Surface Science **603**, 2102 (2009).

[18] R. Bennewitz, M. Bammerlin, M. Guggisberg, C. Loppacher, A. Baratoff, E. Meyer, and H.-J. Güntherodt, *Aspects of Dynamic Force Microscopy on NaCl/Cu(111): Resolution, Tip-Sample Interactions and Cantilever Oscillation Characteristics*, Surf. Interface Anal. **27**, 462 (1999).

[19] C. Loppacher, U. Zerweck, and L. M. Eng, *Kelvin Probe Force Microscopy of Alkali Chloride Thin Films on Au(111)*, Nanotechnology **15**, S9 (2004).

[20] H.-C. Ploigt, C. Brun, M. Pivetta, F. Patthey, and W.-D. Schneider, *Local Work Function Changes Determined by Field Emission Resonances: Na Cl ⁄ Ag ( 100 )*, Phys. Rev. B **76**, 195404 (2007).

[21] Q. Guo, Z. Qin, C. Liu, K. Zang, Y. Yu, and G. Cao, *Bias Dependence of Apparent Layer Thickness and Moiré Pattern on NaCl/Cu(001)*, Surface Science **604**, 1820 (2010).
